# Myh11+ microvascular mural cells and derived mesenchymal stem cells promote retinal fibrosis

**DOI:** 10.1038/s41598-020-72875-x

**Published:** 2020-09-25

**Authors:** H. Clifton Ray, Bruce A. Corliss, Anthony C. Bruce, Sam Kesting, Paromita Dey, Jennifer Mansour, Scott A. Seaman, Christian M. Smolko, Corbin Mathews, Bijan K. Dey, Gary K. Owens, Shayn M. Peirce, Paul A. Yates

**Affiliations:** 1 Department of Biomedical Engineering, University of Virginia, Charlottesville, VA USA; 2 The RNA Institute, University at Albany, State University of New York, Albany, NY USA; 3 Department of Biology, University of Virginia, Charlottesville, VA USA; 4 Robert M. Berne Cardiovascular Research Center, University of Virginia School of Medicine, Charlottesville, VA USA; 5 Department of Molecular Physiology and Biological Physics, University of Virginia, Charlottesville, VA USA; 6 Department of Ophthalmology, University of Virginia, PO Box 800715, Charlottesville, VA 22908 USA

**Keywords:** Mesenchymal stem cells, Retinal diseases

## Abstract

Retinal diseases are frequently characterized by the accumulation of excessive scar tissue found throughout the neural retina. However, the pathophysiology of retinal fibrosis remains poorly understood, and the cell types that contribute to the fibrotic response are incompletely defined. Here, we show that myofibroblast differentiation of mural cells contributes directly to retinal fibrosis. Using lineage tracing technology, we demonstrate that after chemical ocular injury, Myh11+ mural cells detach from the retinal microvasculature and differentiate into myofibroblasts to form an epiretinal membrane. Inhibition of TGFβR attenuates Myh11+ retinal mural cell myofibroblast differentiation, and diminishes the subsequent formation of scar tissue on the surface of the retina. We demonstrate retinal fibrosis within a murine model of oxygen-induced retinopathy resulting from the intravitreal injection of adipose Myh11-derived mesenchymal stem cells, with ensuing myofibroblast differentiation. In this model, inhibiting TGFβR signaling does not significantly alter myofibroblast differentiation and collagen secretion within the retina. This work shows the complexity of retinal fibrosis, where scar formation is regulated both by TGFβR and non-TGFβR dependent processes involving mural cells and derived mesenchymal stem cells. It also offers a cautionary note on the potential deleterious, pro-fibrotic effects of exogenous MSCs once intravitreally injected into clinical patients.

## Introduction

A number of retinal diseases, including epiretinal membranes, proliferative diabetic retinopathy, and proliferative vitreoretinopathy (PVR), have fibrosis as the causative factor in ongoing, progressive vision loss^1^. In particular, PVR is characterized by the presence of pre-retinal and/or sub-retinal fibrous membranes that can interfere with re-apposition of retina to choroid, limiting subsequent recovery of vision^2,3^. Retinal fibrosis occurs following 5–10% of rhegmatogenous retinal detachments, and is a frequent complicating factor associated with ocular trauma and postsurgical re-detachment^4,5^. Pathological fibrosis is largely caused by myofibroblasts, which are activated, contractile fibroblasts that produce excessive amounts of extracellular matrix^6^. In the retina, a number of cell types have been implicated as a putative source of myofibroblasts or fibroblast-like cells, including glial cells^7^, macrophages^8^, and retinal pigment epithelium cells that have detached from the Bruch’s membrane and differentiated into myofibroblasts through epithelial-myofibroblast transition^9^. However, in the absence of cell-specific lineage-tracing, demonstration of the relative contributions of each cell type to retinal fibrosis remains unknown.

Mural cells, specifically microvascular smooth muscle cells (vSMCs) and pericytes (PCs) that reside on the abluminal surface of the microvascular endothelium, are hypothesized to be a population of putative mesenchymal stem cells (MSCs)^10^. In kidney and lung injuries, mural cells, or a subset of these cells, are proposed to exhibit a MSC state and differentiate into myofibroblasts to contribute to the fibrotic pool^11–13^, which commonly leads to scaring, and eventual organ failure. Despite the evidence of potentially deleterious mural cell-to-myofibroblast differentiation in vivo, mural cells are commonly isolated and harvested from the stromal vascular fraction (SVF) of tissues, such as adipose and bone marrow, and used as a therapy in treating a panoply of conditions, with applications in promoting wound healing, stimulating bone replacement, diminishing inflammatory disease, and improving erectile dysfunction^14,15^. However, the cell fate of these injected cells remains largely unexplored, and at times, the delivered MSCs derived from the SVF are not even found after the treated tissue is harvested for analysis^16^. Our limited knowledge of the in vivo cell fate of exogenous MSCs and other cellular components within the SVF very likely contributed to the disastrous and unexpected blinding of multiple patients following injection of the autologous SVF into the vitreous as a treatment for age-related macular degeneration^17^. Within days following injection, patients developed PVR with lens subluxation, retinal detachment, and ultimately loss of vision.

While putative cellular contributors to ocular fibrosis have been identified in the retina, as well as the cornea^18–20^, the potential contribution of mural cells to this process remain largely unknown. Based on the potential for vSMCs and PCs to differentiate into myofibroblasts in other tissues^11–13^, we hypothesize that vSMCs-PCs differentiate into myofibroblasts in the retina and contribute to the pathogenic accumulation of extracellular matrix during the fibrotic process. To test this hypothesis, we traced the Myh11+ mural cell fate using the tamoxifen inducible Cre-recombinase, lineage-tracing mouse model, *Myh11*-CreER^T2^^21^, given Myh11 specificity to only vSMCs and at least a subset of PCs^22^. This study demonstrates Myh11+ mural cells can become classically-defined MSCs, and after severe ocular injury or exogenous intravitreal delivery, mural cells or the derived MSCs contribute to retinal fibrosis. The myofibroblast differentiation of endogenous, retinal Myh11+ mural cells and subsequent generation of fibrotic tissue is attenuated by injection of a small molecule inhibitor for TGFβR. Surprisingly, inhibition of the TGFβ signaling pathway in injected exogenous, Myh11-derived MSCs fails to prevent them from myofibroblast differentiation.

## Results

### Chemical burn induces myofibroblast differentiation of Myh11+ retinal mural cells, resulting in proliferative vitreoretinopathy

**Figure 1:**
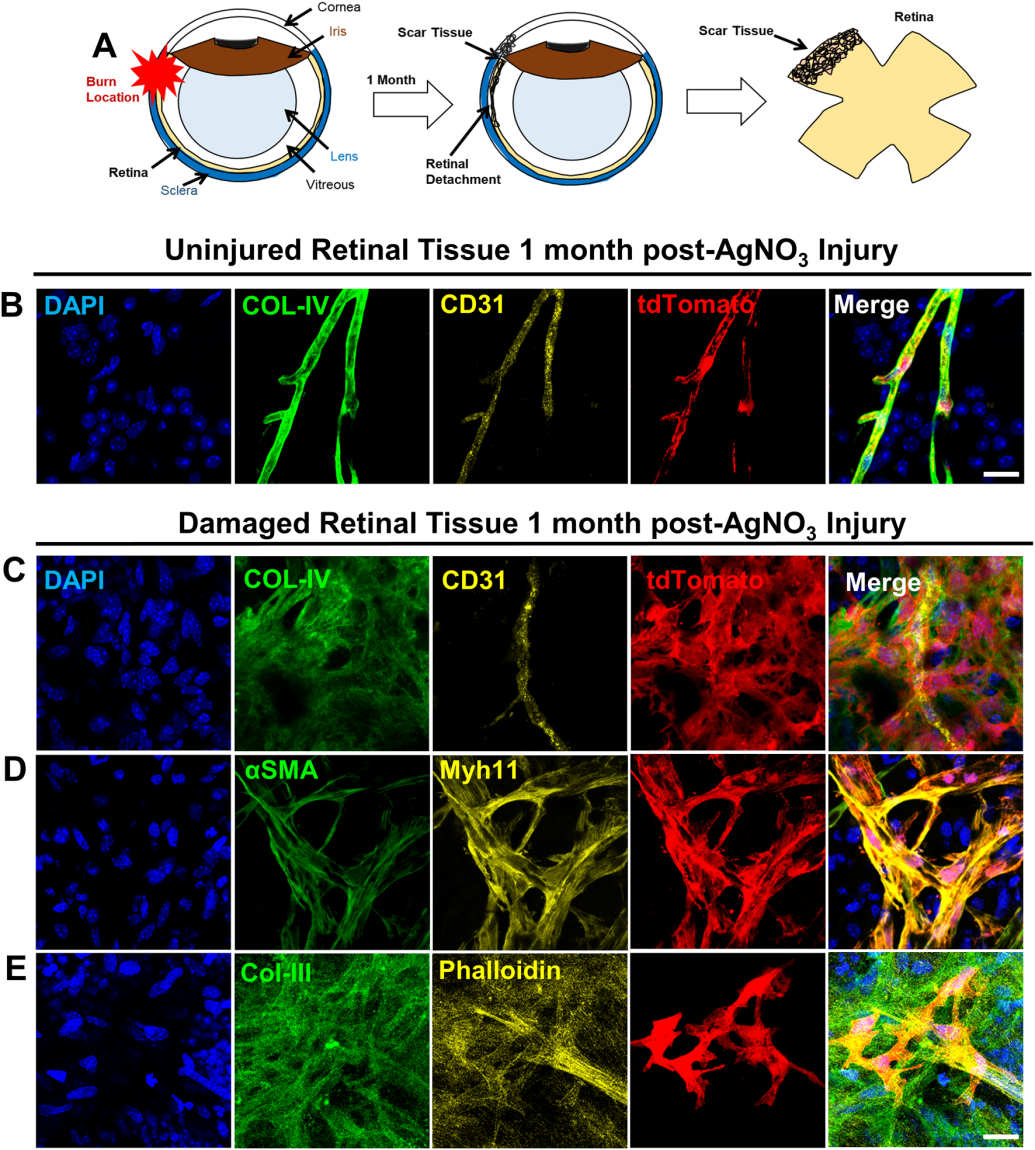
Endogenous Myh11+ mural cells on the retinal microvasculature exhibit a myofibroblast phenotype after a chemical burn to the murine sclera. (**A**) Model demonstrating that silver-nitrate-burn injury to the sclera induced formation of retinal fibrotic scar tissue. (**B**) Immunostained, uninjured retinal tissue revealed Myh11+ mural cells, labeled by tdTomato, are found only on the CD31+ retinal microvasculature. Col-IV is expressed only in the basement membrane of the retinal microvasculature. Scale bar, 15 µm. (**C**) Immunostained retinas one-month post-burn injury showed multiple off-vessel Myh11+ mural cells (tdTomato+), which is indicated by the lack of overlap with the blood vessel endothelium marker CD31. (**D**,**E**) Off-vessel Myh11+ mural cells display αSMA+ stress fibers and are positive for Col-IV, Col-III, and F-actin as shown by fluorescently labeled phalloidin. Scale bar, 15 µm. Animals were tested beginning at 10–12 weeks of age. Immunohistochemistry images are representative of three uninjured and injured eyes of *Myh11*-tdTomato mice. Field of view in injured eyes were selected based on the visual observation of off-vessel Myh11+ mural cells.

Although reports show that perivascular cells can contribute to the fibrotic pool in other end-organs^11–13^, it is unknown whether and to what extent endogenous retinal perivascular cells contribute to fibrotic scaring of the retina. Therefore, we sought to determine if mural cells contribute to retinal fibrosis via myofibroblast differentiation. Myh11 is a contractile protein in the myosin heavy chain family that is expressed by mural cells, particularly vascular smooth muscle cells (vSMCs) and pericytes (PCs) that surround the microvasculature^22,23^. Thus, we used tamoxifen-induced male *Myh11*-CreER^T2^; ROSA26-STOP^FLOX^(tdTomato/tdTomato) (*Myh11*-tdTomato) mice to specifically trace this cell population in the retina during injury. Adopting a severe ocular injury that increases inflammation and retinal cell death^24^, we applied a chemical, AgNO_3_ injury burn to the sclera of tamoxifen-induced male *Myh11*-tdTomato mice (Fig. 1A). Examining the neural retina 1-month post injury, we demonstrate for the first time substantial pre-retinal fibrosis is formed in *Myh11*-tdTomato mice due to the chemical burn. Within the injured tissue, Myh11+ mural cells are dissociated from their underlying CD31+ retinal vasculature and exhibit a myofibroblast morphology. Dissociated Myh11+ mural cells are found amidst pre-retinal fibrotic scar tissue with upregulated expression of αSMA, Col-III, Col-IV, and F-actin (denoted by the staining of phalloidin) (Fig. 1C–E). Interestingly, no tissue-resident fibroblasts were discovered, as no αSMA+/F-actin+ cells were found in the interstitial space of healthy murine retina (Supplementary Fig. S1A–D). This same fibrotic phenomenon was also observed in chemically injured eyes of C57Bl/6J mice (data not shown). No such myofibroblast differentiation is seen in the uninjured eye (Fig. 1B), or even in the injured eye at retinal locations far from the burn site (Supplementary Fig. S1A–D). Myh11 + vSMCs (Supplementary Fig. S1A,D) and PCs (Supplementary Fig. S1B,C) remained on CD31+/Col-IV+ retinal microvasculature with seemingly normal vSMC and PC morphology. In these quiescent retinal regions, Myh11+ mural cells showed no qualitative change in αSMA expression (Supplementary Fig. S1A) as compared to the unburned eye. Quiescent vSMCs and PCs also expressed Myh11, F-actin, and Col-III, with no indication of change in cellular morphology and no migration off the retinal vasculature (Supplementary Fig. S1C,D). These observations demonstrate a traumatic ocular injury, such as a chemical burn, is sufficient to induce endogenous, Myh11+ mural cell migration off retinal vessels, with differentiation into myofibroblasts and subsequent profuse production of Col-IV fibrotic pre-retinal scar tissue.

### Chemical burn upregulates TGFβ and CXCL10, and downregulates multiple interleukins in neural retina

**Figure 2:**
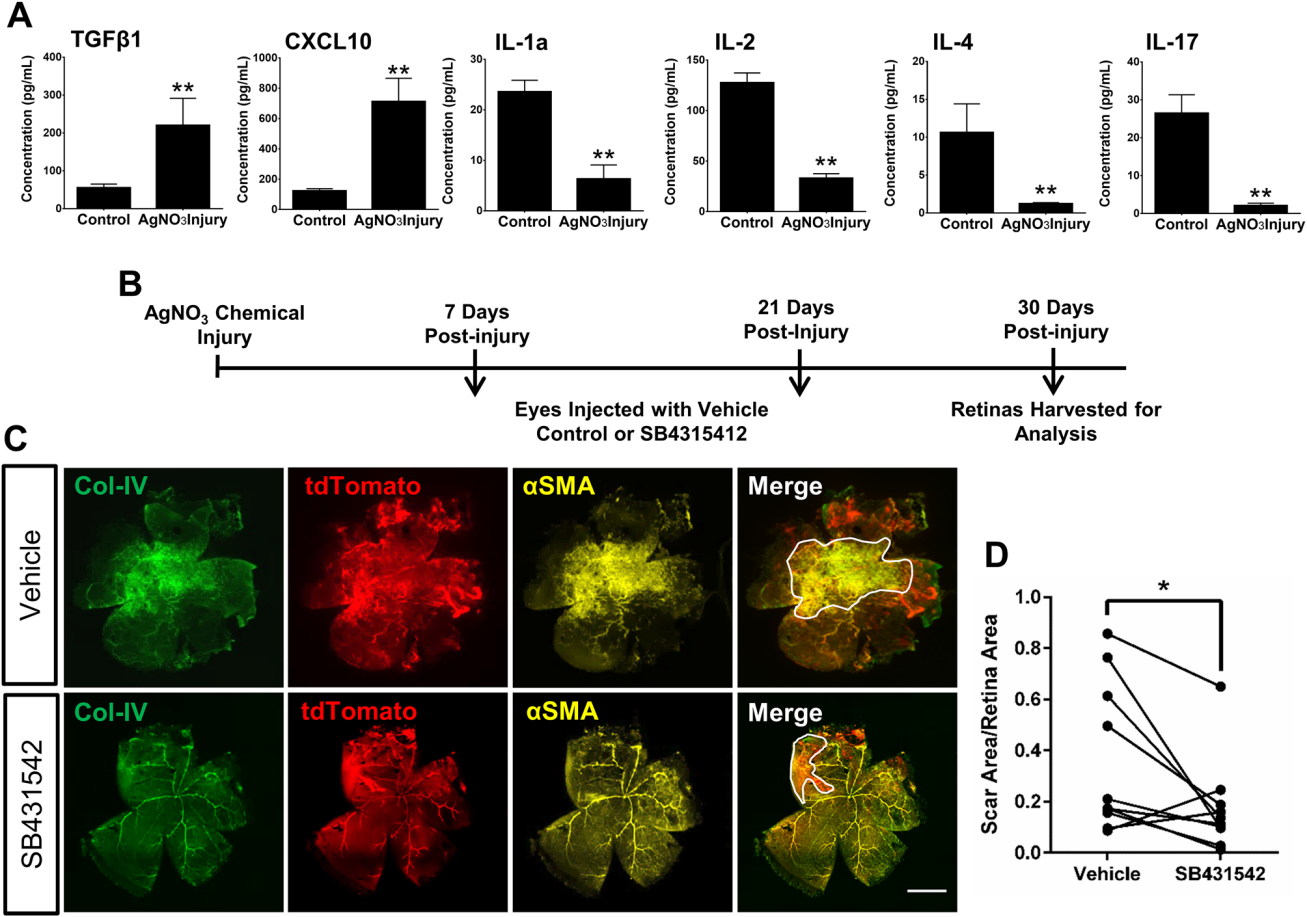
Intravitreal injection of the TGFβR inhibitor, SB431542, reduces retinal scar formation in *Myh11*-tdTomato mice 1-month post chemical burn injury. (**A**) A Luminex multiplex assay demonstrated TGFβ1 and CXCL10 concentrations were significantly increased in injured neural retina of *Myh11-*tdTomato mice 1-month post chemical injury (n = 4 paired eyes). The assay also demonstrated IL-1a, IL-2, IL-4, and IL-17 concentrations were significantly decreased in the neural retina of 1-month-post chemically burned eyes as compared to the contralateral uninjured eyes (n = 4 paired eyes). Data are represented as mean ± standard error of mean (SEM). (**B**) Experimental design for global inhibition of the TGFβ pathway in the eye after chemical-burn injury, where injured eyes received intravitreal injections of either 100 µm SB431542 or carrier control at both 7 and 21 days post injury. (**C**) Representative images display captured tile scans of immunostained retinas harvested 30 days post-injury from eyes intravitreally injected with vehicle control or 100 µm SB431542. Retinal scar formation was revealed by fibrotic Col-IV and αSMA+ stress fibers. Scale bar, 1000 µm. (**D**) Scar area generated by the chemical burn [white outline in (**C**)] was quantified via ImageJ tracing tool, and analysis showed SB431542 significantly decreased retinal fibrosis as compared to contralateral vehicle control injected eyes (n = 10 paired eyes). Animals were tested beginning at 10–12 weeks of age. *p < 0.05, **p < 0.01. Data were analyzed using a ratio paired t test (**A**), or Wilcoxon test (**D**).

The sequence of molecular and cellular events that precipitate fibrosis, such as that which occurs during PVR, remain very poorly understood, and there are a lack of approved molecular therapies for treating this condition^25^. Thus, we next sought to investigate the chemokines and cytokines that may initiate the molecular environment leading to Myh11+ mural cell migration off-vessel and differentiation into myofibroblasts in the retina. A Luminex bead-based multiplex assay on lysed neural retina revealed a significant upregulation of TGFβ1 and CXCL10, paired with a significant reduction in the interleukins IL-1a, IL-2, IL-4, and IL-17 (Fig. 2A). TGFβ1 has a well-described role in activating profibrotic responses and initiating the pathways to promote the increase of stress fibers and collagen secretion^26^, however, its role in promoting Myh11+ mural cell myofibroblast differentiation has not been explored. To validate the potential contribution of TGFβ1 to our observed phenotype, we next inhibited the TGFβR pathway with the small molecule, SB431542, which specifically inhibits the activin type 1 receptors ALK4, ALK5, and ALK7^27^. Intravitreal delivery of 100 µM of SB431542 1-week and 3-weeks post-injury (Fig. 2B) significantly reduced retinal scar area (~ 20%) when compared to burned eyes intravitreally injected with vehicle control (Fig. 2C,D). Thus, the TGFβ pathway appears to play a direct role in formation of fibrosis by promoting off-vessel migration of Myh11+ mural cells and their subsequent differentiation into extracellular matrix producing myofibroblasts.

### Myh11+ mural cells are mesenchymal stem cells (MSCs) in vitro

Although it is demonstrated that Myh11+ mural cells can exhibit a myofibroblast phenotype in vivo, it is unclear if this population represents a potential MSC population based on the definitions of the International Society for Cellular Therapy (ISCT)^28^. According to the ISCT, MSCs derived in vitro are at a minimum to be (1) adherent to plastic; (2) have positive expression for CD73, CD90, CD105, and have negative expression for CD11b, CD19, CD3, CD34, and CD45; and (3) have the capability to differentiate into adipocytes, chondrocytes, and osteocytes. Definitive characterization of a potential MSC phenotype in vitro for mural cells has yet to be accomplished, as prior studies have not used lineage-tracing analyses that allows cell fate of only mural cells to be followed. The reliance of surface marker expression and the lack of lineage tracing studies has made it difficult to conclude mural cells as a definitive source of MSCs.

**Figure 3:**
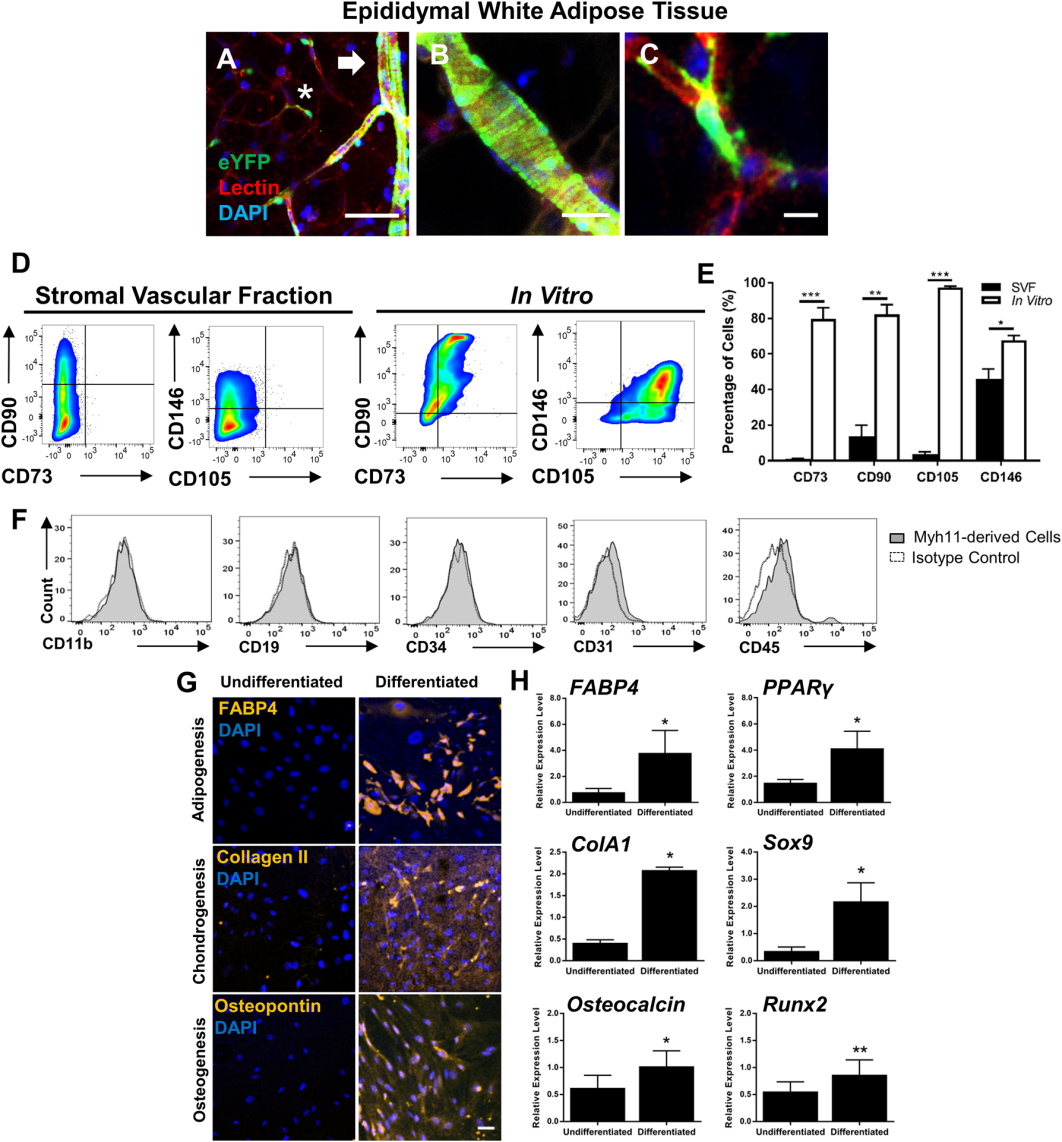
Adipose-derived, lineage-marked Myh11+ mural cells give rise to mesenchymal stem cells (MSCs) during adaptation and growth in vitro*.* (**A**) Immunostained epididymal adipose tissue from *Myh11*-eYFP mice indicated eYFP+ (green) lineage marker is expressed in microvascular smooth muscle cells (vSMCs) (arrowhead) and microvascular pericytes (PCs) (asterisk) along lectin + blood vessels. Scale bar, 50 µm. (**B**) Immunostained adipose tissue revealed vSMC “tire tread” pattern on larger arterioles. Scale bar, 25 µm. (**C**) PCs are wrapped around adipose capillary microvasculature. Scale bar, 10 µm. (**D**) Flow cytometry analysis showed adipose Myh11+ mural cells collected from the SVF have relatively low endogenous expression of CD73, CD90, CD105, and CD146, however, after isolation via fluorescence activated cell-sorting (FACS), cultured, passage 3–5 Myh11+ mural cells significantly increased expression of CD73, CD90, CD105, and CD146 in vitro (three independent flow analyses per panel). (**E**) Graphical representation of flow cytometry analysis demonstrated significant increase of MSC surface antigens in Myh11+ mural cells after isolation from the SVF and cultured in vitro*.* (**F**) Flow cytometry analysis also revealed FAC-sorted and cultured passage 3–5 Myh11+ mural cells lacked expression for hematopoetic, endothelial, and macrophage markers CD11b, CD19, CD34, CD31, and CD45 (three independent flow analyses per panel). (**G**,**H**) Protein and genetic analysis of passage 2 Myh11+ mural cells when cultured in adipogeneic, chondrogenic, or osteogenic media for 14 days. (**G**) Increase in FABP4, Collagen II, and Osteopontin was observed by immunohistochemistry in Myh11+ mural cells undergoing tri-differentiation. Scale bar, 50 µm. (**H**) qPCR showed mRNA expression of protein markers and transcription factors involved in adipogenesis, chondrogenesis, and osteogenesis were significantly upregulated in Myh11+ mural cells following tri-differentiation (n = 3 biological replicates). Relative expression is normalized to GAPDH expression in each sample. Results are represented as mean ± standard error of mean (SEM). Data were analyzed using multiple unpaired t tests followed by the Holm–Sidak post-hoc comparisons to correct for multiple comparisons (**E**), or a ratio paired t-test (**H**). *p < 0.05, **p < 0.01, ***p<0.001. Immunohistochemistry images were captured through randomly sampling of microvasculature tissue and culture wells. Tissue and cultured cells were isolated from *Myh11-*eYFP mice 10–12 weeks of age.

To investigate the MSC characteristics of mural cells, mural cells were analyzed from the epididymal, white adipose tissue of tamoxifen-induced *Myh11*-eYFP mice due to the relative abundance of mural cells in this tissue source. Immunohistochemistry (IHC) revealed that eYFP marked vSMCs and PCs (Fig. 3A–C), demonstrating that vSMCs and PCs in the adipose microvasculature transcribe Myh11 in vivo*.* Importantly, there was no observed labeling of non-perivascular cells with Myh11 by either immunostaining for Myh11 (Supplementary Fig. S2A) or expression of eYFP within the adipose tissue. The endogenous MSC surface antigen profile of mural cells was evaluated by measuring MSC marker expression of uncultured Myh11+ mural cells. After excluding adipose SVF hematopoietic cells and endothelial cells via gating, flow cytometry analysis indicated that Myh11+ mural cells lacked expression for CD73 (0.92 ± 0.40% of gated cells), CD90 (13.71 ± 6.19%), and CD105 (3.69 ± 2.25%) (Fig. 3D). The expression of CD146 is also regarded by studies as a potential perivascular and MSC marker^29,30^. From flow cytometry analysis, approximately 45.94 ± 5.49% of Myh11+ mural cells expressed CD146. Thus, by marker analysis alone, freshly isolated mural cells lack designated in vitro MSC surface markers, and CD146 expression within the adipose Myh11+ population is variable.

To characterize the in vitro MSC profile of Myh11+ mural cells, these cells were isolated from the adipose tissue using FACS based on eYFP expression. Following FACS, sorted cells were cultured on tissue-treated plastic. Performing flow cytometry on these serially passaged cells (of at least two passages), we find significantly increased expression of the designated MSC markers CD73 (79.75 ± 6.09%), CD90 (82.21 ± 5.47%), CD105 (97.35 ± 0.63%), and CD146 (67.73 ± 2.55%) (Fig. 3E). Cultured Myh11+ mural cells also lacked expression (< 3%) for hematopoietic and endothelial cell markers, CD11b, CD19, CD34, and CD45, and the endothelial cell maker CD31 (Fig. 3F). In view of this surface antigen expression, mural cells only acquire a definitive MSC surface marker profile following culture.

The tri-differentiation capability of FAC-sorted, cultured Myh11+ mural cells was investigated by replacing standard growth media with adipogenic, chondrogenic, or osteogenic differentiation media. Immunocytochemistry (ICC) and qPCR confirmed Myh11+ mural cells underwent adipogenesis, chondrogenesis, and osteogenesis following the addition of differentiation media. ICC of cultured cells demonstrated Myh11+ mural cells increased protein expression of FABP4, Col-II, and Osteopontin after exposure to the adipogenic, chondrogenic, and osteogenic media, respectively (Fig. 3G). During adipogenesis, *PPARγ* and *FABP4* mRNA expression is upregulated when compared to undifferentiated cells (Fig. 3H). During chondrogenesis, there is upregulation of *ColA1* and *Sox9* mRNA expression, and during osteogenesis, *Osteocalcin* and *Runx2* mRNA expression levels are also increased. Thus, by the ISCT criteria, Myh11+ mural cells are putative MSCs.

### Intravitreally injected MSCs derived from Myh11+ mural cells contribute to murine retinal fibrosis

The injection of adipose-derived MSCs are considered a therapeutic for regenerative medicine due to their immomudalation and pro-angiogenic paracrine profile, as well as their ability to provide juxtacrine support for endothelial cell angiogenic networks^31–34^. However, the intravitreal injection of presumed adipose-derived MSCs resulted in blinding age-related macular degeneration patients through the development of PVR^17^. Therefore, we sought to rigorously explore the cell fate of intravitreally injected Myh11-derived MSCs in a retinopathy model, specifically the oxygen-induced retinopathy (OIR) model, and access the impact of MSCs on retinal angiogenesis and potential fibrosis. In the OIR model, the central retinal microvasculature is ablated by exposure to hyperoxia from post-natal day 7–12 (P7–P12)^35^. After returning to normoxia, retinal blood vessels undergo neovascularaziation similar to what is found in ocular vasculopathy diseases such as late-stage, proliferative diabetic retinopathy.

**Figure 4:**
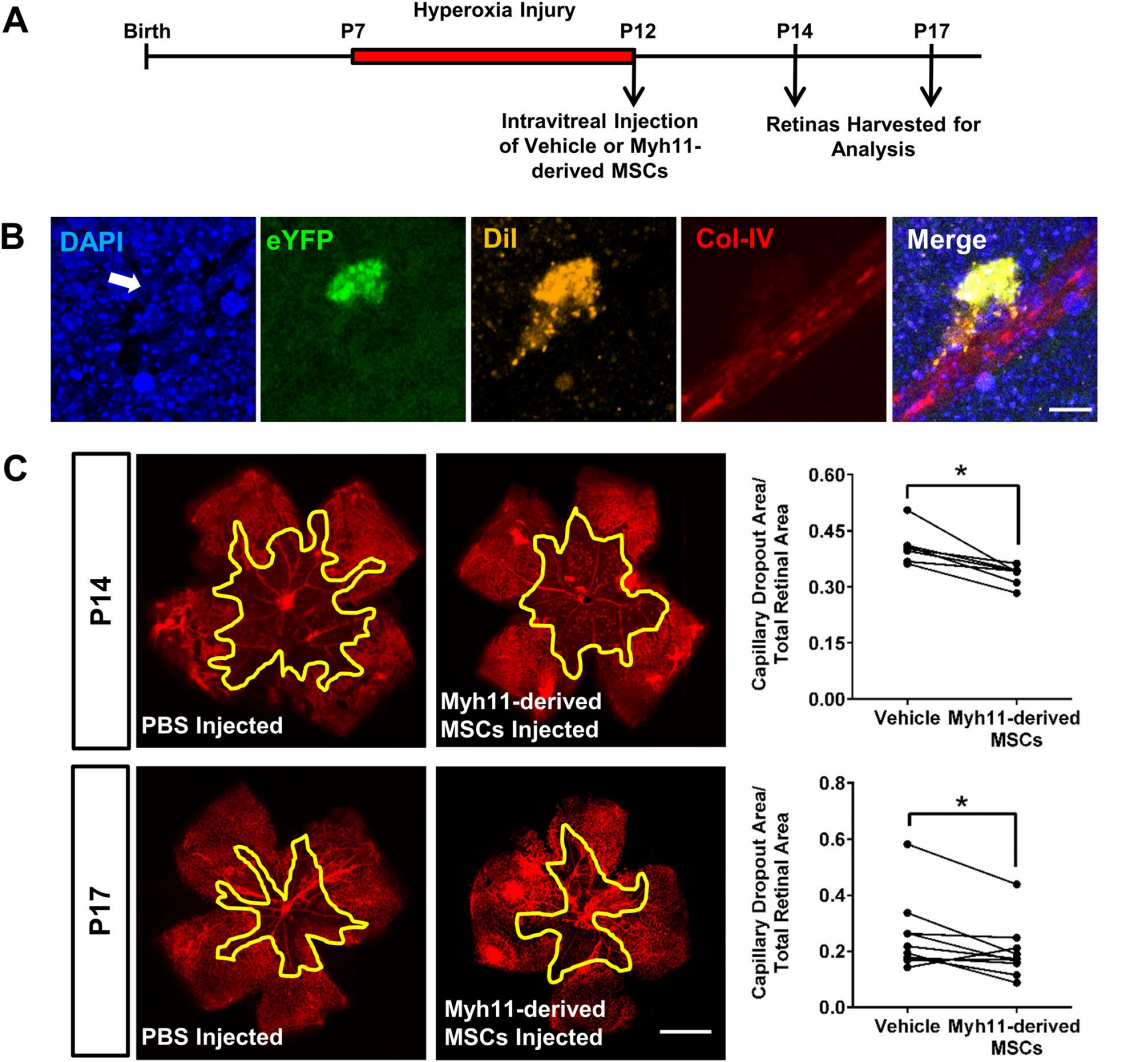
Intravitreally injected Myh11-derived MSCs accelerate microvasculature recovery and adopt a perivascular position during murine oxygen-induced retinopathy (OIR). (**A**) Diagram illustrating the timeline of the murine OIR model and intravitreal injection of Myh11-derived MSCs. After hyperoxia injury from P7 to P12, pup eyes were intravitreally injected with PBS-vehicle or 10,000 Myh11-derived MSCs at passage 3–5, and analyzed at P14 and P17 post-injection. (**B**) At P14 and P17, intravitreally injected Myh11-derived MSCs (DiI+/eYFP+) were able to integrate into the retinal tissue and associate with Col-IV+ retinal vasculature. Scale bar, 10 µm. (**C**) Representative immunostained retinal flatmounts at P14 and P17 are shown with outlined area (yellow) representing capillary dropout region caused by OIR. Retinal blood vessels were immunostained with lectin (red). Quantification of capillary dropout in ImageJ showed eyes intravitreally injected with Myh11-derived MSCs experienced a significant reduction in capillary dropout area at P14 (n = 7 paired eyes), and at P17 (n = 10 paired eyes) when compared to the contralateral PBS vehicle control eyes. Scale bar, 1000 µm. *(p < 0.05). All data were analyzed using a Wilcoxon test.

After mice experienced hyperoxic exposure from P7 to P12, 10,000 cultured MSCs derived from Myh11+ mural cells were intravitreally injected into the eyes of P12 mice. At P14 and P17, the retinas were harvested and analyzed using IHC to observe cell fate and associated retinal vasculature changes (Fig. 4A). Confocal analysis demonstrated injected Myh11-derived MSCs are found in perivascular positions, with a typical phenotypic appearance as endogenous retinal PCs (Fig. 4B). At P14, 1.54 ± 0.34% of the Myh11-derived MSCs were integrated into the retinal tissue, with 38.14 ± 16.06% of these cells adopting a perivascular position. At P17, there was an increase in both the number of integrated cells (2.91 ± 0.96%) as well as their propensity to adopt a perivascular position (58.24 ± 8.46%). Regarding the retinal vasculature, we observed at P14, eyes that were injected with Myh11-derived MSCs had a significant reduction (18.4 ± 0.19%, n = 7) in avascular area compared to PBS-injected contralateral eyes (Fig. 4C). By P17, there was a significant reduction (22.6 ± 0.03%, n = 10) in retinal capillary dropout compared to PBS-injected contralateral eyes (Fig. 4C). These results suggest injected Myh11-derived MSCs can re-adopt a mural cell position consistent with their cell origin.

**Figure 5:**
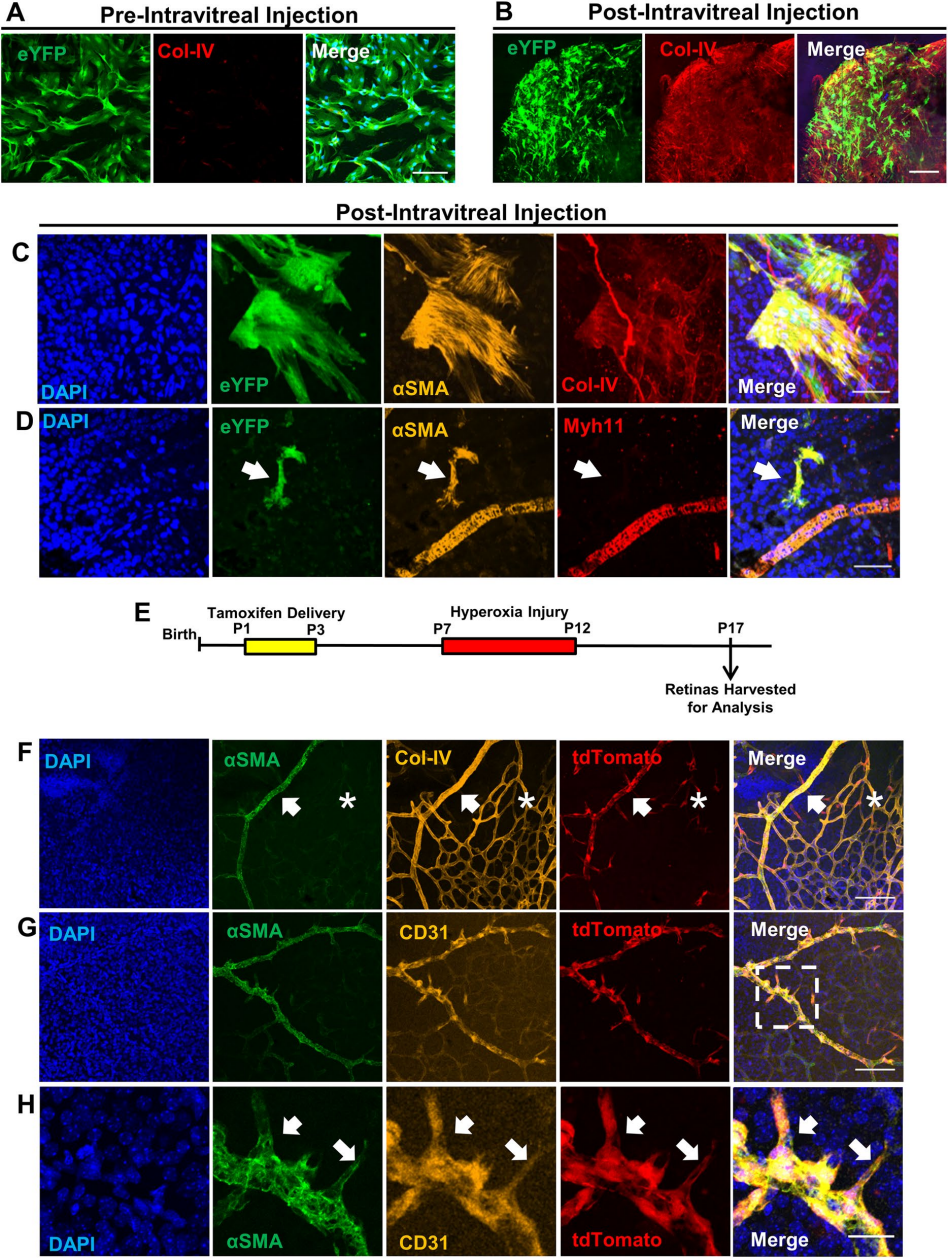
Within the murine OIR model, intravitreal injected Myh11-derived MSCs in the vitreous gel exhibit a myofibroblast phenotype, while endogenous, retinal Myh11+ mural cells remain in a perivascular position. (**A**) Immunostained Myh11-derived MSCs lacked expression of Col-IV in vitro, however, (**B**) immunostained retinas revealed intravitreal injected Myh11-derived MSCs expressed Col-IV in the vitreous gel, which formed a dense, fibrotic pre-retinal membrane in murine OIR eyes. Scale bars, 200 µm. (**C**) Intravitreal injected passage 3–5 Myh11-derived MSCs expressed αSMA+ stress fibers and Col-IV, and (**D**) Myh11-derived MSCs have reduced expression of Myh11 following injection (arrow) compared to the endogenous Myh11 expressed in retinal mural cells. DAPI stained nuclei of underlying retinal ganglion cells in addition to injected MSCs. Scale bars, 100 µm. (**E**) Experimental design where tamoxifen is delivered postnatal day 1–3 *Myh11-*tdTomato mice to induce expression of tdTomato in Myh11+ mural cells. Induced mice are then exposed to hyperoxia from postnatal day 7–12 to cause OIR injury, with retinas harvested at P17 to determine cell fate of endogenous, retinal Myh11+ mural cells. (**F**) At P17, endogenous, retinal Myh11+ mural cells resided on Col-IV+/CD31+ vessels, with αSMA expression higher in vSMCs (arrow) than PCs (asterisk). Scale bar, 100 µm. (**G**) Myh11+ mural cells remained on vessel with no vSMCs-PCs found off vessel. Scale bar, 100 µm. (**H**) Neither retinal vSMCs or PCs extended processes from CD31 tip cells (arrow) at the leading front of the regenerating retinal microvasculature. Scale bar, 25 µm. Immunohistochemistry images represent fields of view that were sampled based on the presence of eYFP and tdTomato expression within culture and tissue. Images are also representative of at least three biological replicates or animals.

However, the majority of the intravitreally injected MSCs do not invest within the retina tissue. Thus, we next assessed the fate of the intravitreally injected mural cell-derived MSCs within the vitreous of OIR eyes, which is located above the neural retina. Col-IV labeling revealed a substantial increase in extracellular matrix production of the injected cells remaining in the vitreous, with formation of a fibrotic pre-retinal membrane that is reminiscent of PVR (Fig. 5A,B). Prior to intravitreal injection, IHC confirmed that Myh11 was expressed in both vSMCs and PCs (vSMC-PCs) (Supplementary Fig. S2A), while αSMA, is predominately expressed by vSMCs as compared to PCs (Supplementary Fig. S2B). Once FAC-sorted from the adipose tissue, cultured Myh11+ mural cells were positive for both Myh11 and αSMA stress fibers (Supplementary Fig. S2A–C), but acquired low expression in Col-IV (Fig. 5A). Unfortunately, we discovered that injected Myh11-derived MSCs remaining in the vitreous exhibited αSMA stress fibers, and have decreased Myh11 expression as compared to endogenous, retinal vSMCs and PCs (Fig. 5C,D). The presence of aSMA+ stress fibers, disassociation from the retinal vasculature in a diseased environment, and Col-IV expression^26,36^ suggests that MSCs adopt a default myofibroblast phenotype when injected within the vitreous cavity of murine OIR eyes.

### Endogenous retinal Myh11+ mural cells do not differentiate into myofibroblasts in OIR

Given that intravitreally injected Myh11-derived MSCs differentiate into myofibroblasts in the murine OIR model, we next examined if there were similar differentiation of endogenous, retinal Myh11+ mural cells following OIR injury. To explore this hypothesis, we tamoxifen-induced cre-recombinase in P1 to P3 *Myh11-*tdTomato mice using intragastric tamoxifen injections. From P7 to P12, the tamoxifen-induced *Myh11*-tdTomato mice were introduced to hyperoxia, and at P12 these mice were returned to normoxia and retinas analyzed at P17 (Fig. 5E). As indicated by the tdTomato expression, no endogenous, retinal Myh11+ mural cells were observed off-vessel with Col-IV + matrix production and/or an obvious myofibroblast morphological phenotype directly above or within the retinal vascular plane (Fig. 5F). In the area of central retinal capillary dropout, Myh11+ mural cells remained directly in contact with CD31+ blood vessels and CD31+ tip cells extended towards the interstitial space of the retina (Fig. 5G,H). Taken together, the data revealed that injected Myh11-derived MSCs promoted retinal vasculature growth and reintegration with the retinal vasculature to likely serve as functional vSMCs or PCs. However, injected Myh11-derived MSCs remained off vessel and differentiated into myofibroblasts with copious production of Col-IV matrix characteristic of PVR. For endogenous Myh11+ mural cells, this differentiation was not observed within the same retinal injury model. It remains unclear the cause of this distinct difference in behavior, though it is tempting to speculate that association with and integration into the retinal vasculature may fundamentally modulate cell behavior away from myofibroblast differentiation.

### Decreasing Smad4 in Myh11-derived MSCs does not decrease Col-IV secretion after injection into OIR mice

**Figure 6:**
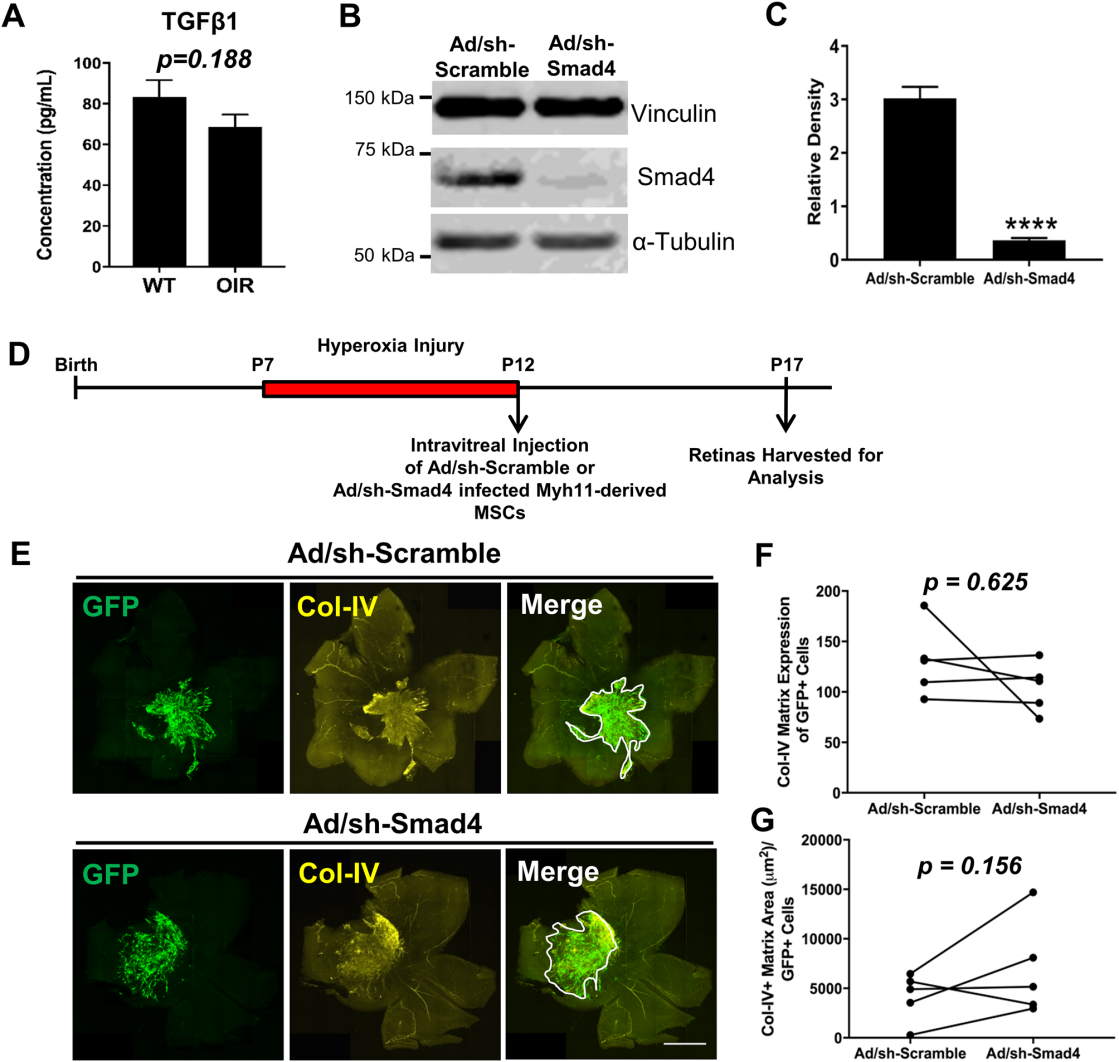
Smad4 knockdown within Myh11-derived MSCs does not abolish induction of proliferative vitreoretinopathy following intravitreal injection of these cells within the murine OIR model. (**A**) A Luminex multiplex assay demonstrated no significant difference in active TGFβ1 protein concentration within the neural retina and vitreous of P14 wildtype (WT) mice and WT mice that underwent OIR (n = 5 unpaired eyes). Cropped Western blot from representative lanes of one gel (**B**) and densitometry quantification (**C**) demonstrated significant knockdown of Smad4 in Myh11-derived MSCs through mSmad4-shRNA adenovirus vectors (n = 6 biological replicates). Data are represented as mean ± standard error of mean (SEM). (**D**) Experimental design illustrating the intravitreal injection of Smad4-shRNA infected Myh11-derived MSCs versus Scramble-shRNA infected Myh11-derived MSCs at P12 following OIR injury, with subsequent harvest of injected retinas at P17. (**E**) Representative tile scan images of immunostained retinas revealed Col-IV pre-retinal matrix production (white outline in Merge panels) in eyes of P17 OIR mice intravitreally injected at P12 with passasge 6–8 Myh11-derived MSCs infected with either Ad-GFP-U6-mSmad4-shRNA or with Ad-GFP-U6-scramble. Fibrotic scar indicated by Col-IV is evident in both eyes regardless of Smad4 knockdown. Scale bar, 1000 µm. (**F**) No significant difference is found in fibrotic scar Col-IV matrix expression of the eyes intravitreally injected with Myh11-derived MSCs infected with Ad-GFP-U6-scramble adenovirus vectors (GFP+) as compared to Myh11-derived MSCs infected with Ad-GFP-U6-mSmad4-shRNA adenovirus vectors (GFP+) (n = 6 paired eyes). (**G**) No significant difference is found in fibrotic scar Col-IV matrix area normalized by the number of GFP+ cells found within this fibrotic scar. ****p < 0.0001. Data were analyzed using unpaired t test (**A**, **C**), or Wilcoxon test (**F**, **G**).

We next sought to determine if TGFβ signaling pathway may also regulate differentiation of intravitreally injected Myh11+ mural cell derived-MSCs into myofibroblasts. Surprisingly, no significant difference was found in active TGFβ1 concentration measured in retina and vitreous samples of age-matched OIR mice and wildtype normoxia C57Bl/6J mice (Fig. 6A). Despite equivalent levels of TGFβ1 in the retinal microenvironment, it is known the TGFβ pathway can also potentially be activated via mechanotransduction^37^, as well as positive feedback loops from other pathways, including the WNT signaling pathway^38^. Thus, we attempted to directly block TGFβ signaling in Myh11-derived MSCs by cell-specific knockdown of Smad4 using shRNA-expressing adenovirus vectors. Our hypothesis was that reduction of Smad4, would lead to a decrease in Col-IV+ matrix production in these cells once intravitreally injected into the OIR model. Two days pre-intravitreal injection, Myh11-derived MSCs (derived from *Myh11*-tdTomato mice) were infected at 3000 MOI of Smad4-shRNA or scramble-shRNA adenovirus vectors, with co-expression of GFP indicating successful infection (Supplementary Fig. S2A,B). Smad4 knockdown in Myh11-derived MSCs was confirmed through western blot before intravitreal injection (Fig. 6B,C, Supplementary Fig. S4). After two days of culturing with adenovirus vectors, 10,000 MSCs were injected in P12 OIR mice and retinas were harvested at P17 to investigate the Col-IV matrix production (Fig. 6D,E). Interestingly, Col-IV matrix expression level was unchanged by Smad4 knockdown as compared to the scramble control (Fig. 6F). Col-IV matrix area was not found significantly affected, however, there was an unexpected, trending increase in the Col-IV matrix area per GFP+ cells in eyes injected with Smad4-knockdown MSCs (Fig. 6G). These results suggest that while TGFβ signaling appears to be significantly involved in myofibroblast differentiation of endogenous, retinal Myh11+ vSMCs-PCs, non-TGFβ dependent pathways may be activated during myofibroblast differentiation of Myh11-derived MSCs following intravitreal injection.

## Discussion

Retinal fibrosis remains an unfortunate complication of several retinal diseases, including diabetic retinopathy, ocular trauma, and retinal detachment, for which no clinically proven treatments are currently available. The pathophysiology underlying retinal fibrosis remains complex, with multiple cell types appearing to contribute to the fibroblastic phenotype characteristic of this scarring process, making it difficult to determine if a single targeted cell or molecular pathway can ameliorate disease. The primary question we sought to address was whether and to what extent endogenous retinal mural cells may contribute to this process, given that they are presumed central to development of fibrosis in other end-organs, but have received less attention in retinal fibrotic disease. The secondary question we sought to address, given the putative identification of mural cells as a source of adult MSCs in adipose tissue, was how mural cell-derived MSCs may have contributed to the blinding of age-related macular degeneration patients when injected exogenously into the vitreous^17^. Answering both questions was aided by our use of a defined, lineage-tracing murine model to track the cell fate of Myh11+ mural cells, specifically microvascular smooth muscles (vSMCs) and perictyes (PCs). Our study is the first to utilize mural cell lineage tracing to elucidate their contribution to retinal fibrotic disease.

We discover after chemical injury to the murine eye that lineage-labeled Myh11+ vSMCs-PCs are capable of disembarking from the underlying injured retinal vasculature and phenotypically switching to myofibroblasts that produce copious collagen, a *sine qua non* of proliferative vitreoretinopathy (PVR)^39,40^. This severe injury also creates a highly inflammatory environment as demonstrated by substantial upregulation of cytokines, chemokines, and growth factors in the affected retinal tissue. Surprisingly, Myh11-derived myofibroblasts maintain expression of Myh11, which challenges the claimed specificity of Myh11 to vSMCs and PCs^41^, but adds to our recent observations that Myh11 is also expressed by corneal endothelial cells^42^. Whether myofibroblast Myh11 remains of functional importance will require determining Myh11 protein turnover and synthesis in the Myh11-derived myofibroblasts, and assessing its role in myofibroblast contractility.

Despite myofibroblast differentiation at the site of injury, this differentiation remains localized as Myh11+ mural cells distant from the injury site appear to retain normal cell morphology attached to their underlying vessel. One possible interpretation among several is that the blood vessel injury must be severe and the local molecular milieu must have a specific composition, including a significant concentration of factors such as matrix metalloproteinases, to disrupt the vSMC and PC connection with their underlying microvascular basement membrane and endothelial cells. Once off-vessel, myofibroblast differentiation of mural cells appears to be a default phenotype. These contrasting results prototypically demonstrate that retinal fibrosis is a multifaceted disease with Myh11-derived myofibroblasts likely functioning in concordance with multiple ocular cell types to form the resultant vision-impairing fibrotic membranes.

A potential role for TGFβ in regulating PVR has been examined in multiple animal and human studies^43,44^, but remains unclear. TGFβ1 is directly implicated in the subsequent precipitation of systemic fibrosis in other tissue such as the lung, kidney, and liver^45,46^. Despite this, elevated TGFβ1 levels are not detected in the vitreous of human PVR patients^47^. In contrast, and more in line with non-ophthalmic reports, our chemical injuries demonstrate active TGFβ1 present in lysates of murine neural retina. While the source of TGFβ1 remains unknown, it would not be surprising if the elevated concentration resulted from the activation of Müller glial cells, given their demonstrated high expression of multiple cytokines and inflammatory factors in isolates taken from human PVR patients^48^. The exact mechanisms by which TGFβ1 may contribute to mural cell to myofibroblast transformation also remains unknown, and could include both direct and indirect pathways. As discussed in fibrotic kidney disease^49^, we hypothesize that the increase of TGFβ1 in the neural retina may lead to vasoconstriction, which in turn, activates retinal Myh11+ mural cells to differentiate into myofibroblasts.

By targeting TGFβR via the small molecule inhibitor, SB431542, we present a possible clinical approach to reduce or eliminate at least some aspects of retinal fibrosis. If proven, this would be a substantial advancement as the only available treatment for retinal fibrosis is painstaking surgical microdissection. Our results demonstrate intravitreal delivery of SB431542 following chemical injury preserves the retinal microvasculature and attenuates Myh11+ mural cell migration off-vessel to differentiated myofibroblasts. In this model, active TGFβ1 expression level remains elevated in injured retina, but it appears that SB431542 inhibition of TGFβR is able to prevent the activation of downstream pro-fibrotic pathways that results from binding of TGFβ1. It remains unclear if the observed effect of SB431542 is secondary to its direct effect on TGFβR expressed on Myh11+ mural cells, or if it due to more general inhibition of TGFβR on multiple cell types in the retina. Future work specifically targeting TGFβR inhibition in Myh11+ mural cells may help elucidate the role of this pathway in the mural cell-to-myofibroblast differentiation, including possible induction of intermediary cell types in this differentiation process.

There remains controversy about whether and to what extent mural cell differentiation occurs in vivo, as in multiple injury and aging models, there were no observed differentiation or even off vessel lineage-marked Tbx18+ perivascular cells^50^. In contrast, prior studies have demonstrated Myh11+ mural cells possess the ability to differentiate in vivo into beige adipocytes under cold exposure^51^, and into macrophage-like pro-inflammatory foam cells in atherosclerosis^52–54^. We now add to this list by demonstrating myofibroblast differentiation of Myh11+ mural cells, and suspect in fact it may be a common differentiation path for these cells after severe tissue injury.

It has been suggested, but not yet demonstrated, that this discrepancy in in vivo mural cell and perivascular cell differentiation is perhaps the consequence of the chosen lineage marker or injury model used in these studies^55^. In addition, the field is further confounded by recent single cell RNAseq data showing the existence of multiple subtypes of mural cells that appear to differ between vascular beds^56^. This seems to follow the general principle that a single marker does not solely define a specific subpopulation of cells, and conforms to the observation that no single marker appears to label all perivascular cells. Therefore, future investigation exploring the role of mural cell differentiation and other terminal cell types within retinal and systemic fibrosis, will require consistency and rigor in regards to the injury model, selected timepoints, selected lineage marker or sets of markers, and chosen pre-clinical animal model.

We further demonstrate for the first time isolated, adipose Myh11+ mural cells meet all specified in vitro ISCT criteria required for MSC classification^28^. Specifically, Myh11+ mural cells adhere to plastic, upregulate the expression of classically-defined MSC surface markers, and possess the ability to tri-differentiate in vitro under the appropriate media conditions. We show in adipose tissue that Myh11 expression is found only on vSMCs and PCs, eliminating the possibility of confounding cell types isolated from this specific tissue. We further show cultured Myh11+ mural cells maintain expression of Myh11 and αSMA, and are capable of being followed through multiple passages with continued expression of a fluorescent reporter. Cultured Myh11+ mural cells do continue to express Myh11 once injected intravitreally, but at significantly reduced level compared to Myh11 expression on adjacent endogenous retinal mural cells. The reason for this decrease remains unclear, but possible contributors include multiple serial passaging in culture as well as differentiation to a MSC phenotype. Surprisingly, freshly, isolated Myh11+ mural cells have very low expression of the requisite ISCT MSC surface markers. Conversely, these surface antigens are upregulated in culture, which suggests that the artificial culture conditions may stimulate the transformation to an MSC phenotype.

While we can define requisite marker expression in vitro to confirm that Myh11+ mural cells can assume an MSC identity, it remains unclear whether expression of these markers are necessary or sufficient to permit differentiation of Myh11+ mural cells to alternate cell types in vivo. Our results suggest, but do not prove, that divestment from the vasculature, rather than MSC marker expression, is a critical, in vivo step for Myh11+ mural cell differentiation. This of course raises the even more controversial issue of whether in vivo differentiation from Myh11+ mural cell to myofibroblast or alternate cells types requires the mural cell to first assume an intermediate MSC identify before the differentiation process can occur. Unfortunately, this is beyond the scope of the present work, though we can comment that attempting to find this MSC state in vivo, if it exists, may prove difficult, as there is substantial overlap in surface antigen expression between perivascular cells and the putative MSC population^29,57,58^.

We demonstrate that a large majority of intravitreally injected MSCs derived from Myh11+ mural cells differentiate into myofibroblasts in the oxygen-induced retinopathy (OIR) model, with abundant production of Col-IV+ pre-retinal scar tissue. This occurs despite their lacking significant Col-IV expression in vitro*.* Note that the OIR model was utilized not as a model of retinal fibrosis (it is not), but rather because we had previously demonstrated that intravitreally injected, adipose-derived MSCs can migrate to the retina and re-associate with the retinal vasculature with expression of standard pericyte markers^59^. The problem we observed in this report is that the majority of injected Myh11-derived MSCs remain in the vitreous, and this subpopulation of injected cells exhibit a myofibroblast phenotype. This result again suggests that anchoring of the injected MSCs to the retinal vasculature may prevent myofibroblast differentiation, and that a key step in inhibiting some aspects of retinal fibrosis may be to maintain pericyte-vessel association.

In view of our current results and the evident fibrosis in the multiple patients injected with adipose SVF, we further suggest this myofibroblast differentiation is a likely contributing cause for their resulting blindness^17^. We cannot of course rule out additional contributing factors, including substantial induction of inflammation due to the injected cells^60^, presence of fibroblasts and other inflammatory cell types within the SVF, or digestion of ocular structures from retained collagenase in the SVF. Nevertheless, it is clear that MSCs derived from culturing Myh11 mural cells appear primed for generating retinal fibrosis, and may well be sufficient in of themselves to account for the evident PVR in these patients.

Since TGFβ1 levels were not significantly upregulated in the oxygen-induced retinopathy murine retinal environment in which Myh11-derived MSCs were injected, we can only speculate as to the other factors contributing to this myofibroblast differentiation in the eye. Prior studies suggest that a 3D fibril matrix environment is sufficient to initiate myofibroblast activation through mechanotransductive pathways, resulting in the increase of stress fibers and collagen production^61,62^. Therefore, the 3D collagen matrix of the vitreous body^63^, where most of the injected cells reside, is likely a contributing factor in generating the myofibroblast phenotype of the injected MSCs. Although sub-retinal injection of these stem cells is a viable alternate approach for delivery, there remains similar concerns regarding the risk of fibrosis or other adverse side effects^64^. Our findings argue that future clinical use of adipose-derived MSCs must pay particular attention to the differentiated phenotype of injected adipose MSCs themselves, rather than principally focus on their secondary paracrine effects on surrounding tissues^14^. Despite the aforementioned complications, the opportunity to replace damaged or lost perivascular cells on damaged retinal microvasculature, or perhaps even facilitate growth of new retinal vessels, using adipose-derived MSCs remains an intriguing possibility.

Although, a small percentage of Myh11-derived MSCs were able to relocate to a perivascular position, future studies should explore how to stimulate the injected MSCs to adopt a mural cell phenotype within the tissue since this seems essential to achieving therapeutic efficacy rather than deleterious complications. We are surprisingly not able to significantly attenuate myofibroblast differentiation of injected adipose Myh11-derived MSCs despite SMAD inhibition, and this differentiation notably occurs in an ocular injury environment having relative normal TGFβ1 levels. Interestingly, SMAD inhibition has produced similar failures in reducing systemic fibrosis, despite the presumed role of TGFβ in regulating this process^65,66^. As previously mentioned, TGFβ1 may assume a larger role in initiating off-vessel transition of mural cells, while subsequent fibrotic transformation of off-vessel mural cells may be initiated through additional fibrotic regulatory pathways, including YAP/TAZ, BMP, MRTF, and WNT^67^. In fact, we recently reported dual SMAD inhibition in Myh11-derived MSCs can promote a corneal endothelial cell differentiation in vitro^42^*.* Thus, future studies will require a more systematic approach to regulate the molecular milieu and multiple potential fibrotic signaling pathways of Myh11-derived MSCs. We believe insights gained by such refined studies can inform our understanding of systemic fibrosis found in any number of disease states, as our results indicate these processes are inextricably intertwined.

## Methods

### Experimental mouse models

All animal studies were approved by the University of Virginia’s Animal Care and Use Committee and adhered to the ARVO Statement for the Use of Animals in Ophthalmic and Vision Research*. Myh11*-CreER^T2^ mice were crossed with ROSA26-STOP^FLOX^(eYFP/eYFP) (The Jackson Laboratory, stock number 006148) and ROSA26-STOP^FLOX^(tdTomato/tdTomato) (The Jackson Laboratory, stock number 007914) to generate *Myh11*-CreER^T2^;ROSA26-STOP^FLOX^(eYFP/eYFP) (*Myh11*-eYFP) and *Myh11*-CreER^T2^;ROSA26-STOP^FLOX^(tdTomato/tdTomato) (*Myh11*-tdTomato) mice. To induce cre-recombinase activity, 6–8-week-old mice were intraperitoneally injected daily over the course of 10 days with 0.1 mg of tamoxifen (Sigma-Aldrich, Cat#T5648) diluted in 100 μL of peanut oil. All adult male mice received a total of 1 mg of tamoxifen during the course of the 10-day injection period. Adult mice were analyzed or used for experimentation 2–4 weeks after the last tamoxifen injection to insure proper clearance, and rule out the possibility that other unmarked cell types transiently express Myh11 in inflammation and acquire fluorophore expression without being of vSMC-PC lineage.

For OIR experiments, late-stage pregnant C57Bl/6 females (gestational days 11–15) were purchased from The Jackson Laboratory (stock number 00664). At postnatal day 1 (P1) to P3, *Myh11-*tdTomato mice received intragastric injections of 50 µg tamoxifen in 50 µL peanut oil using the previous protocol^68^ to label Myh11-lineage vSMCs-PCs before the start of the OIR model. Once mice were at the age of P7, the mother and mice were both exposed to hyperoxia to induce OIR injury as described below.

### Primary cell cultures

All primary cells were isolated from white, epididymal adipose tissue and were seeded at an original density of 1.5 × 10^4^ cells/cm^2^ on tissue culture-treated plastic. Cells were cultured in DMEM media supplemented with 10% FBS and 1% antibiotic/antimycotic. Cells were passaged using StemPro Accutase Cell Dissociation Reagent (ThermoFisher, A1110501) after reaching 70–80% confluency and media was changed every 2–3 days. All cells analyzed and intravitreally injected throughout the study were between passage 2 and passage 8.

### Immunohistochemistry (IHC) and immunocytochemistry (ICC)

Epididymal white adipose tissue was harvested from tamoxifen-induced male *Myh11*-eYFP mice and fixed by submersion in 4% paraformaldehyde (PFA) for at least 12 h in 4 °C. After fixation, tissue was permeabilized with 0.3% Triton X-100. Tissue was later blocked for 3 h at room temperature with mouse, donkey, or goat serum to prevent nonspecific secondary antibody. Retinas, the retinal pigment epithelium layer, and sclera were harvested from enucleated eyes of male *Myh11*-tdTomato mice and C57Bl/6J mice, and fixed in 4% PFA for 1 h at room temperature. All ocular tissue was then permeabilized with 0.3% Triton X-100 for 1 h at room temperature, and blocked with serum for 1 h at room temperature.

Cultured cells were washed and fixed with 4% PFA for 1 h at room temperature, and permeabilized with 0.3% Triton X-100 for 5 min at room temperature. Following permeabilization, cells were blocked with serum for 1 h at room temperature. After permeabilization and blocking steps, all tissue and cell samples were than washed, and labeled with isolectin GS-IB_4_ (ThermoFisher, I32450, 1:200 dilution) or phalloidin (ThermoFisher, A22287, 1:200 dilution) depending on the experimental design. Tissues were stained with primary antibodies for GFP (ThermoFisher, A-21311, 1:200 dilution), Myh11 (Kamiya Biomedical Company, MC-352 1:400 dilution), RFP (Abcam, AB62341, 1:200 dilution), Col-III (Abcam, AB7778, 1:100), Col-IV (Bio-Rad, 134001, 1:200 dilution), CD31 (BioLegend, 102504, 1:200 dilution), and/or αSMA (Sigma-Aldirch, C6198 and F3777, 1:400 dilution). Non-conjugated primary antibodies were labeled with the appropriate secondary antibodies: donkey anti-rabbit Alexa Fluor 546 (ThermoFisher, A10040, 1:650 dilution), goat anti-rat Alexa Fluor 568 (ThermoFisher, A-11077, 1:650 dilution), donkey anti-goat Alexa Fluor 647 (ThermoFisher, A-21447, 1:650 dilution), and donkey-rabbit Alexa Fluor 647 (ThermoFisher, A-31573, 1:650 dilution). Tissues and cells were preserved and nuclei were stained with VECTASHIELD Antifade Mounting Medium with DAPI (Vector Laboratories, Burlingame, CA). Image acquisition was performed on whole mounted tissue and cultured cells using a Zeiss LSM 880 confocal microscope, and images were processed using ImageJ (NIH, Bethesda, Maryland, USA, https://imagej.nih.gov/ij/).

### Flow cytometry characterization

White, epididymal adipose tissue from *Myh11*-eYFP mice was enzymatically digested in 4 units/mL Liberase (Sigma-Aldrich, 5401119001) and 0.74 units/mL of elastase (Worthington, LS002279) within DMEM/F12 media for 1.5 h at 37 °C. After enzymatic digestion, mature adipocytes were removed by centrifuging the collected digested mixture at 1100 rpm for 5 min, followed by the discardment of the supernatant. The remaining pellet, or SVF, was washed and red blood cells were removed using red blood cell lysis buffer (ThermoFisher, 00-4333-57). Fc receptors of remaining SVF cells were blocked with antibodies to CD16/CD32 (ThermoFisher, 14-0161-81, 1:500 dilution). After blocking, cells were stained with primary antibodies to CD11b (BD Bioscience, 553309, 1:200 dilution), CD19 (BD Bioscience, 553784, 1:200 dilution), CD31 (eBioscience, 13-0311-811, 1:200 dilution), CD34 (BD Bioscience, 560518, 1:200 dilution), CD45 (BD Bioscience, 553078, 1:200 dilution), CD73 (BD Bioscience, 561543, 1:2000 dilution), CD90 (SouthernBiotech, Birmingham, AL, 1740-09, 1:400 dilution), CD105 (BD Bioscience, 564746, 1:400 dilution), and CD146 (BD Bioscience, 562232, 1:400 dilution). Biotin conjugated antibodies to mark hematopoietic cell and endothelial cell markers (CD11b, CD19, CD31, CD34, and CD45) were labeled with streptavidin PE-Cy5.5 (ThermoFisher, SA1018, 1:1000 dilution) to establish a “negative dump” and exclude hematopoietic cells and endothelial cells from gating analysis. To determine cell viability, LIVE/DEAD Fixable Aqua Dead Cell Stain Kit (ThermoFisher, L34957) was used according to the manufacturer’s protocol. Fluorescence minus one (FMO) controls were used to determine correct expression of CD73, CD90, CD105, and CD146.

Flow cytometry was performed on FAC-sorted, cultured primary cells, where the FAC-sorting protocol is described in the next methods section. To perform flow cytometry characterization on cultured cells, cells were uplifted with StemPro Accutase Cell Dissociation Reagent (ThermoFisher, A1110501). Fc receptors were blocked with serum, and cells were stained with the above primary antibodies to CD19, CD31, CD45, CD73, CD90, CD105, and CD146. Primary antibodies to CD11b, CD19, CD31, CD34, and CD45 were labeled with goat anti-rat AlexaFluor 546 (ThermoFisher, A-11077, 1:400 dilution). Cells were labeled with DAPI (ThermoFisher, D1306, 1:1000 dilution) to only include viable cells throughout the analysis. Cells were also labeled with isotype control antibodies IgG2a (BioLegend, 400501, 1:200 dilution) IgG2b (BioLegend, 400601, 1:200 dilution), and IgGc (BioLegend, 400701, 1:200 dilution) to serve as negative controls and distinguish between positive and negative gating for CD11b, CD19, CD31, CD34, and CD45. All flow cytometry characterization was performed on a BD LSRFortessa with DIVA 6.0. Flow cytometry data was analyzed in FlowJo v10 and FCS Express 6.0.

### Fluorescence activated cell sorting

To extract Myh11+ mural cells for culturing, white, epididymal adipose tissue from *Myh11*-eYFP and *Myh11*-tdTomato mice was enzymatically digested as previously described^59,69^. Briefly, epididymal adipose tissue was enzymatically digested at 37 °C for 1 h in 1 mg/mL in collagenase type I (ThermoFisher, 17100017) digestion buffer consisting of 200 nM adenosine, 2.5% (w/v) bovine serum albumin, 20 mM HEPES (4-(2-hydroxyethyl)-1-piperazineethanesulfonic acid), 1.2 mM monopotassium phosphate, 4.7 mM potassium chloride, 1.2 mM magnesium sulfate heptahydrate, 120 mM sodium chloride, and 1.3 mM calcium chloride dehydrate. After 1 h digesting, mature adipocytes were removed by centrifuging the collected mixture at 1100 rpm at 5 min and discarding the supernatant. The pellet, or SVF, was suspended in red blood cell lysis buffer (ThermoFisher, 00-4333-57) for 5 min at room temperature. Next, cells were suspended and washed in DMEM, and filtered through a 70-µm and 40-µm mesh. Collected cells were resupsended in FACS Buffer consisting of DMEM, 50% BSA, 5 mM EDTA, and DAPI (ThermoFisher, D1306, 1:1000 dilution). Myh11+ mural cells were FAC-sorted using a BD Influx Cell Sorter. Cell populations were immediately sorted into DMEM media supplemented with 10% FBS and 1% antiobiotic/antimycotic, and plated and cultured as described above.

### In vitro tri-differentiation assay

For the tri-differentiation assay, cultured cells were introduced to standard low glucose media, adipogenic, chondrogenic, or osteogenic media according to the manufacturer’s protocol (R&D Systems, SC010). After 14 days cultured under the appropriate differentiation media, mRNA expression was analyzed using qPCR. Immunocytochemistry was also performed following the steps mentioned above to stain for primary antibodies to FAPB4, Col-II, osteopontin (1:200 dilution) following the manufacturer’s protocol (R&D Systems, SC010). Primary antibodies for protein detection were labeled with secondary antibodies donkey anti-rabbit AlexaFluor 647 (ThermoFisher, A31573, 1:400 dilution) and donkey anti-sheep AlexaFluor 647 (ThermoFisher, A21448, 1:400 dilution).

### Collection of RNA and qPCR

Before and after tri-differentiation, RNA samples were collected from all cell populations to measure gene expressions of transcription factors and proteins involved in differentiation. RNA was isolated using an RNeasy MicroKit (Qiagen), and cDNA was synthesized using an iScript cDNA Synthesis Kit (Bio-Rad) or Superscript IV Reverse Transcriptase (ThermoFisher). iQ SYBR Green Supermix (Bio-Rad) and SensiMix II Probe Kit (Bioline) was used as detection kits, and samples were analyzed on a CFX96 Touch (Bio-Rad). GAPDH was used as the housekeeping gene throughout analysis. Primer sequences can be found in Supplementary Table S1.

### Oxygen induced retinopathy (OIR)

The OIR model was adapted as previously described^59^ and followed the guidelines of the ARVO Statement of the Use of Animals in Ophthalmology and Vision Research. C57Bl/6J mothers and mice were immersed in a closed chamber supplied with 75% oxygen from postnatal day 7 (P7) to postnatal day 12 (P12). Following OIR, eyes of the P12 mice were injected with 10,000 Myh11-derived MSCs in 1.5 µL of PBS. Contralateral eyes were injected with equal volume of 1.5 µL PBS to serve as the appropriate control. To confirm signal in the retinal tissue, cultured MSCs were labeled with Vybrant DiI Cell-Labeling Solution (ThermoFisher) to assist with tracking and counting injected cells that integrated in the retinal tissue and made contact with the retinal vasculature. To analyze the retinal vasculature structure and the tissue integration of injected cells, mice were euthanized at P14 and P17 and retinas were harvested and labeled with isolectin GS-IB_4_, and primary antibodies to αSMA, Myh11, and Collagen-IV as described earlier in the above methods section. Tile scan and z-stack confocal images were captured of entire retinas to measure capillary dropout area and retina. Capillary dropout was defined as areas within the retinal tissue lacking tertiary vasculature structures. Capillary dropout area and retina area was calculated using ImageJ tracing tool. The capillary dropout area was normalized by the retina area for statistical analysis. To observe the scar formation of Myh11-derived MSCs above the superficial microvascular plexus, the vitreous gel was also harvested and wholemounted in tandem with the retina and further processed for IHC.

Myh11-derived MSCs infected with adenovirus vectors as described below were washed with PBS before injection into OIR mice. 10,000 infected Myh11+ MSCs were intravitreally injected into P12 OIR mice in a volume of 1.5 µL of PBS. Five days after injection at P17, retinas and scar tissue were harvested and stained for DAPI, Col-IV, and GFP as described above in the IHC and ICC methods.

### Sclera chemical injury burn

Silver nitrate burns were applied to the sclera near the boundary of the cornea of anesthetized tamoxifen-induced 12–14-week-old *Myh11*-tdTomato mice. While mice were under isoflurane anesthesia, proparacaine hydrochloride ophthalmic solution drops were applied to the eyes to serve as a topical anesthetic. After applying the topical anesthetic, the eyes were proptosed, and two silver nitrate burns were placed for approximately 2 s within a single 1–2 mm region of the sclera, or the region of the eye directly under the limbal vessels. Buprenex was intraperitoneally injected immediately after the burn injury to serve as an analgesic. Seven days and 21 days post-injury, TGFβR inhibitor, SB431542 (Sigma-Aldrich, S4317), was intravitreally injected into the eye at 100 μM in 1.5 μL of 0.3% (v/v) DMSO in PBS. Contralateral eyes received equal volume of 1.5 μL of 0.3% (v/v) DMSO in PBS to serve as a control for eyes injected with 100 μM SB431542. One month-post silver nitrate, eyes were enucleated, and stereoscope images were captured to determine eye measurements. The retinas were harvested, and labelled with phalloidin and stained with primary antibodies targeting αSMA, Col-III, Col-IV, CD31, and RFP as described above in the earlier methods section. Confocal tile scans and z-stack images were captured of the entire retina to calculate retina area and scar area, which was defined as the area of retina that contained off-vessel Myh11+ myofibroblasts (marked by αSMA+ stress fibers) within a Col-IV matrix. The scar area was normalized by retinal area for further statistical analysis.

### Adenoviral shRNA infection of Myh11+ MSCs

The shRNA adenovirus vector Ad-GFP-U6-mSMAD4-shRNA (Cat#ADV-272602) and a non-specific scrambled shRNA adenovirus vector (Cat#1122) was purchased from Vector Biolabs (Malvern, PA, USA). Both adenovirus vectors contained a GFP reporter gene under control of the U6 promoter, thus we used Myh11-derived MSCs derived from *Myh11*-tdTomato male mice. MSCs were transfected at 50–3000 MOI for 48 h in standard media culture conditions described above. Infected MSCs were also lysed for quantitative fluorescent immunoblotting as described below.

### Immunoblotting

Quantitative fluorescent immunoblotting was performed as previously described^70^. Protein was collected from samples using RIPA lysis buffer and later prepared in 40 µL of dithiothreitol-containing Laemmli sample buffer. Samples were electrophoresed in 10% polyacrcylamide gels with tris-gylcine running buffer (25 mM tris base, 250 mM glycine, and 0.1% SDS) at 130 V for 1 h. Proteins were transferred to a PVDF membrane (Millipore) in transfer buffer (25 mM tris, 192 mM glycine, 0.037% SDS, and 10–40% methanol) at 100 V for 1 h on ice. PVDF membranes were blocked with 0.5X Odyssey blocking buffer (LI-COR) + TBS + 0.1% Tween-20. Primary antibodies were used to recognize the following proteins: Vinculin (Millipore, #05-386, 1:10,000 dilution), alpha-Tubulin (Abcam, #89884, 1:20,000 dilution), and SMAD4 (Cell Signaling Technology, #46535, 1:10,000 dilution). After incubation in primary antibodies overnight at 4 °C, membranes were washed and probed with secondary antibodies diluted with 0.5 × Odyssey blocking buffer. The following secondary antibodies were used to target the above primary antibodies: IRDye 680LT goat anti-mouse (LI-COR, #926-68020, 1:20,000 dilution), IRDye 680LT donkey anti-chicken (LI-COR, #926-68028, 1:20,000 dilution), and IRDye 800CW goat anti-rabbit (LI-COR #926-32211, 1:20,000 dilution). Membranes were scanned on an Odyssey infrared scanner (LI-COR) at 169-µm resolution and 0-mm focus offset. The ban intensities of the scanned 16-bit images were quantified by densitometry in ImageJ.

### Luminex analysis

Thirty days post-chemical injury burn described above, the eyes were enucleated from the mice, and the neural retina was harvested and placed in 40 µL of RIPA lysis and extraction buffer (ThermoFisher, 89900). The samples were kept on ice until ultrasonicated for 2 min. Ultrasonicated samples were then centrifuged at 18,000*g* at 4 °C for 15 min. The supernatant was collected and analyzed through a custom Luminex MAGPIX bead-based multiplex panel to measure active TGFβ1, CXCL10, IL-1a, IL-2, IL-4, and IL-17.

### Statistical analysis

All statistical tests were performed in GraphPad Prism version 7.00 (GraphPad Software, La Jolla, California, USA, www.graphpad.com). Multiple unpaired t tests were used to compare two mean values of unpaired samples. A ratio paired t test was used to compare the two mean ratio values of paired samples following normal distribution. A Wilcoxon test was used to compare to the two mean values of paired samples that were not normally distributed which was determined by Shapiro–Wilk normality test. Significance for all test was defined as p < 0.05. One asterisk represents p < 0.05, two asterisks represent p < 0.01, three asterisks represent p < 0.001, and four asterisks represent p < 0.0001.

### Image acquisition and analysis

Laser scanning confocal microscopy of cultured cells and tissue samples was performed using a Carl Zeiss LSM 880 confocoal microscope (air objectives 10 × EC Plan-Neofluar with NA 0.30, 20 × Plan-Apochromat with NA 0.8, oil objectives 40 × Fluar with NA 1.30 and 63 × Plan-Apochromat DIC M27 with NA 1.4) with multichannel scanning. ZEN Imaging software (Carl Zeiss) was used for image acquisition. Random sampling was performed on individual in vitro biological replicates. Micrographs were rendered to maximum intensity projections, and quantitative analysis was performed using ImageJ software. Microsoft PowerPoint version 16.0.0 (Redmond, Washington, USA https://www.microsoft.com) was used to prepare manuscript figures and illustrations.

## Supplementary information

Supplementary Information.

## Data Availability

All the data are included in the main text or in the Supplementary Materials. Paul Yates (University of Virginia, Charlottesville, Virginia) is the lead contact and responsible for all reagent and resource requests. Please contact Paul Yates at pay2x@virginia.edu for all inquiries.
