# Supplementary material for: Myh11+ microvascular mural cells and derived mesenchymal stem cells promote retinal fibrosis
Source: Sci Rep. 2020 Sep 25;10:15808. doi: 10.1038/s41598-020-72875-x (PMC7519078; doi:10.1038/s41598-020-72875-x)
Supplement: Supplementary file 1 — Supplementary Information. [file 41598_2020_72875_MOESM1_ESM.pdf]

Myh11+ microvascular mural cells and derived mesenchymal stem cells promote retinal fibrosis

Authors

H Clifton Ray[1†] Bruce A Corliss[1†], Anthony C Bruce[1], Sam Kesting[1], Paromita Dey[2], Jennifer Mansour[3], Scott A Seaman[1], Christian M Smolko[1], Corbin Mathews[1], Bijan Dey[2], Gary K Owens[4,5], Shayn M Peirce[1], Paul A Yates[1,6*]

Affiliations

[1]Department of Biomedical Engineering, University of Virginia, Charlottesville, Virginia, United States of America
[2]The RNA Institute, University at Albany, State University of New York, Albany, New York, United States of America
[3]Department of Biology, University of Virginia, Charlottesville, Virginia, United States of America
[4]Robert M. Berne Cardiovascular Research Center, University of Virginia School of Medicine, Charlottesville, Virginia, United States of America
[5]Department of Molecular Physiology and Biological Physics, University of Virginia, Charlottesville, Virginia
[6]Department of Ophthalmology, University of Virginia, Charlottesville, Virginia, United States of America

[†]Both authors contributed equally to this work

[*]Contact Information for Corresponding Author:

Paul A. Yates, MD, PhD,
University of Virginia Dept. of Ophthalmology
PO Box 800715
Charlottesville, VA 22908
Phone: 434-924-5485
Fax: 434-924-5180
Email: pay2x@virginia.edu

This Supplement contains:
Supplement Figures 1-4

Supplemental Figures

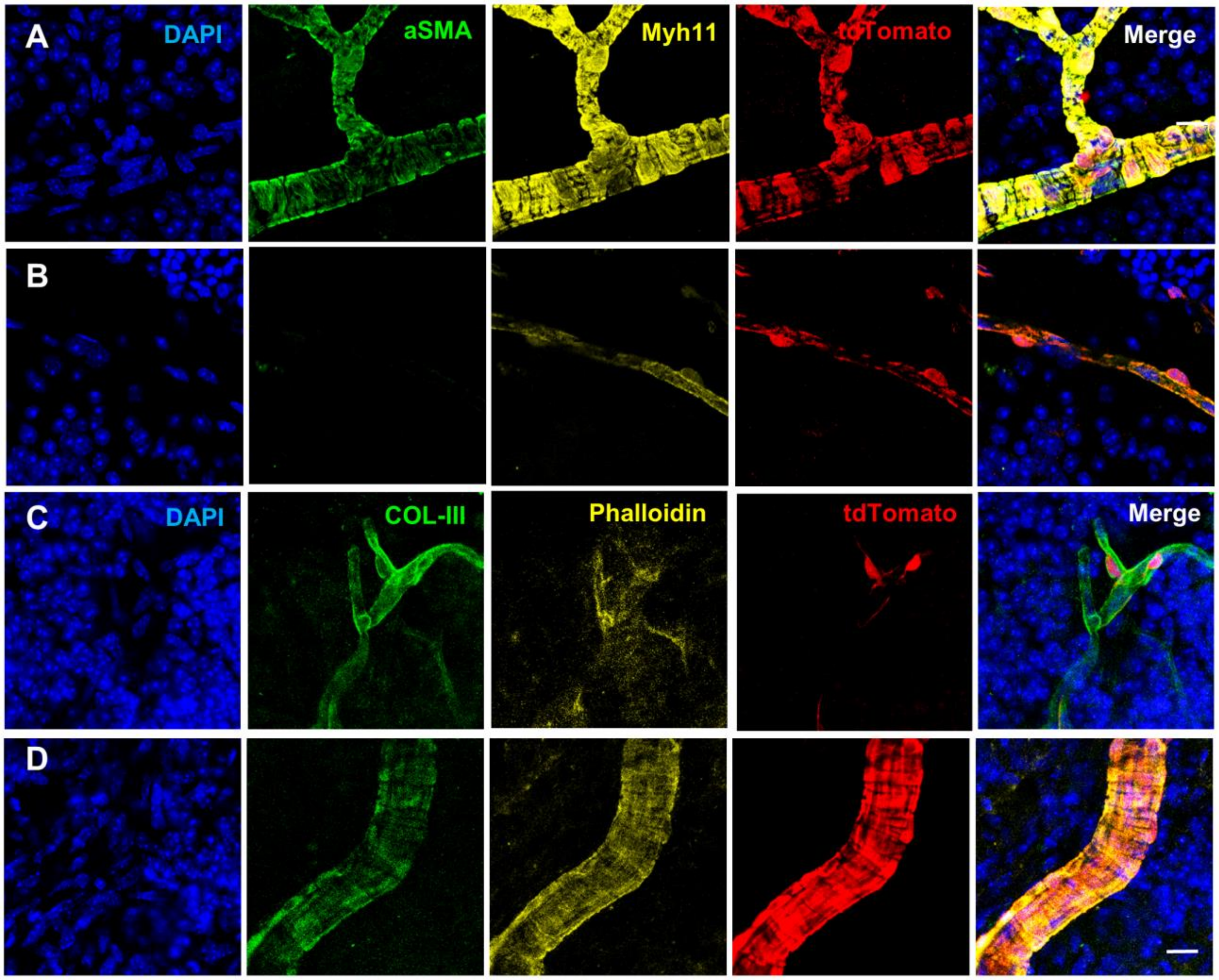

Figure S1. Related to Figure 1. Myh11+ microvascular smooth muscle cells (vSMCs) and pericytes (PCs) remain fully associated with retinal microvasculature at retinal locations distant from the scleral burn.

(**A-D**) Immunostained retinas revealed Myh11+ vSMCs and PCs (tdTomato+) cells are located in the hemi-retina opposite from the scleral burn site and remain fully invested on large retinal arteriolar blood vessels (**A**) and capillaries (**B**) and express the Myh11 protein. These quiescent lineage-traced vSMCs were also positive for Col-III, F-actin (Phalloidin+) and αSMA, while lineage-traced PCs did not express F-actin or αSMA. Scale bar: 15 μm in (**A-D**). Images are

representative of random sampling of the uninjured region of retinas isolated from injured mice at 10-12 weeks of age (n=3).

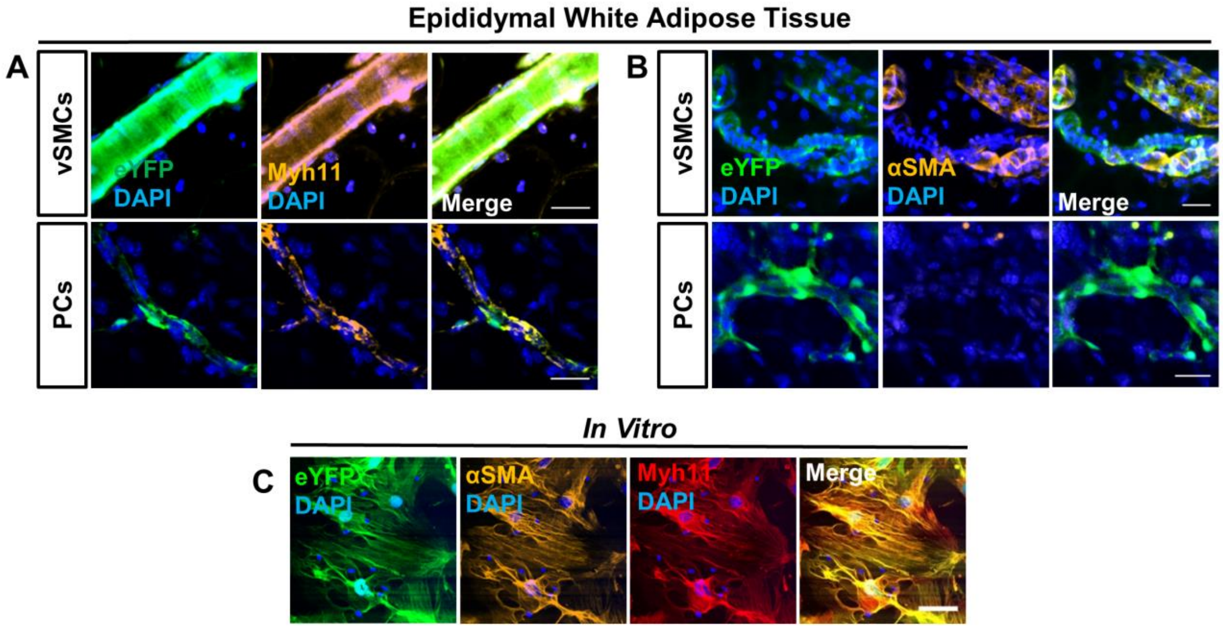

Figure S2. Related to Figure 3 and 5. ***In vivo*** **and** ***in vitro*** **expression of αSMA and Myh11 in Myh11+ vSMCs-PCs.** (**A-B**) Representative images of epididymal, white adipose tissue from tamoxifen-induced *Myh11*-eYFP+/+ mice immunostained for Myh11 and αSMA. Myh11 expression overlapped with, but was not entirely coincident, with eYFP expression in both vSMCs and PCs. In contrast, αSMA expression was higher in vSMCs on arterioles. (**C**) FAC-sorted and cultured Myh11+ vSMCs-PCs remained fluorescent for the eYFP lineage marker and expressed both αSMA (99.66% ± 0.24%, n=3 biological samples) and Myh11 (98.62%±0.42%, n=3 biological samples) at passage 5 *in vitro*. Before culture, Myh11+ mural cells were found to represent 3.28±0.32% (n=8 biological samples) of cells in the adipose stromal vascular fraction (SVF). Scale bars: 50 µm (top) and 25 µm (bottom) panels in (**A,B**) and 100 µm in (**C**). Animals were tested at 10-12 weeks of age (**A,B**). Results are represented as mean ± standard error of mean (SEM). All images are representative of three animals and biological replicates, and fields of view were selected based on random sampling of microvasculature tissue and cell culture wells.

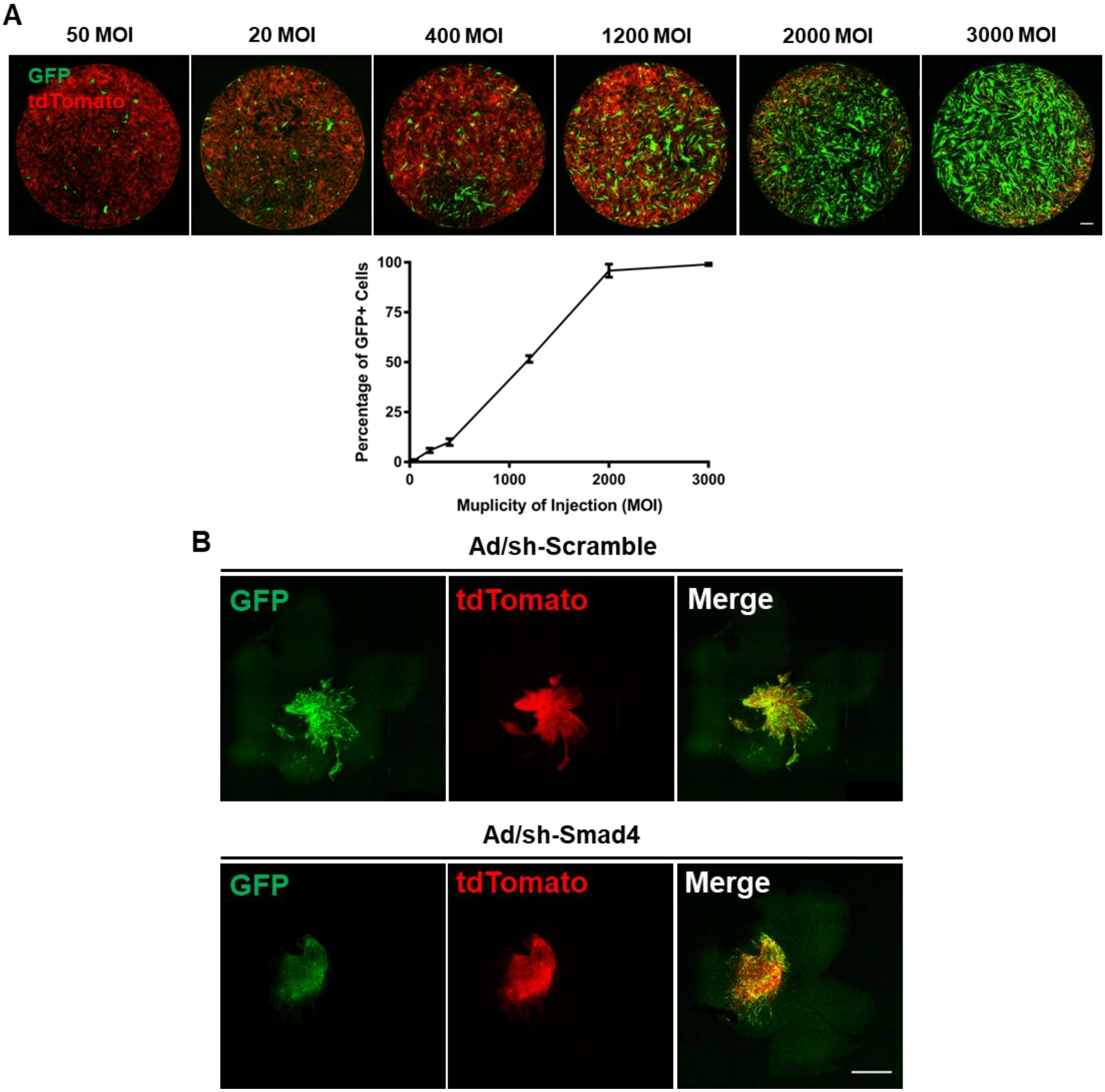

Figure S3. Related to Figure 6. Myh11-derived MSCs are efficiently infected with shRNA adenovirus vectors, resulting in expression of GFP marker. (**A**) GFP expression of passage 6-8 Myh11-derived MSCs infected with 50-3000 MOI of Ad-GFP-U6-mSmad4-shRNA. Percentage of GFP+ cells increased with MOI. Data are representative of three independent

biological replicates. (**B**) tdTomato and GFP expression are both maintained in intravitreally injected Myh11-derived MSCs (TdTomato+) infected with either Ad-GFP-U6-scramble-shRNA or Ad-GFP-U6-mSmad4-shRNA prior to injection. Images represent tile scan of entire retina wholemount. Scalebars: 500 µm in (A) and 1000 µm in (B).

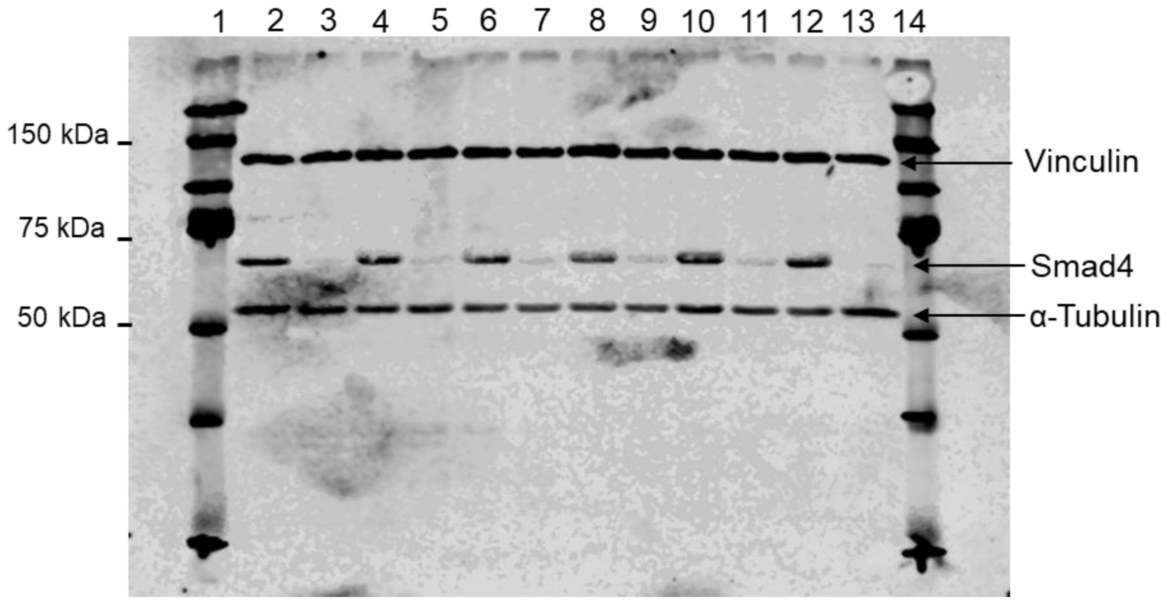

Figure S4. Related to Figure 6. Full-length Western blot to confirm MSCs derived from Myh11+ mural cells were efficiently infected with shRNA adenovirus vectors to knockdown Smad4.

Lanes 1 and 14: Ladder.

Lanes 2, 4, 6, 8, 10, and 12: Individual biological replicates (n=6) of Myh11-derived MSCs infected with 3000 MOI of Ad-GFP-U6-scramble.

Lanes 3, 5, 7, 9, 11, and 13: Individual biological replicates (n=6) of Myh11-derived MSCs infected with 3000 MOI of Ad-GFP-U6-mSmad4-shRNA.

Lanes 6 and 7 were used as representative images for Figure 6.

Table S1. List of primers used for qPCR within *in vitro* MSC tri-differentiation experiments.

**Gene**	**Sequence**
FABP4	F: AGCTTGTCTCCAGTGAAAACTTCG<br>R: CATTTACGCTGATGATCATGTTGG
PPARγ	F: GGATAAAGCATCAGCCTTCCACT<br>R: TCCGGCAGTTAAGATCACACCTA
COLA1	F: AATGAAGAACTGGACTGTCCCAAC<br>R: GGTCCCTCGACTCCTACATCTTCT
SOX9	F: AAGAAAGACCACCCCGATTACAA<br>R: AGCGCCTTGAAGATAGCATTAGG
Osteocalcin	F: GACTCGGATGAATCTGACGAATCT<br>R: GACCTCAGTCCATAAGCCAAGCTA
Runx2	F: GAACCAAGAAGGCACAGACAGAA<br>R: AGGCGGGACACCTACTCTCATAC
